# A Nonredundant Role for the TRPM6 Channel in Neural Tube Closure

**DOI:** 10.1038/s41598-017-15855-y

**Published:** 2017-11-15

**Authors:** Yuko Komiya, Zhiyong Bai, Na Cai, Liping Lou, Namariq Al-Saadi, Courtney Mezzacappa, Raymond Habas, Loren W. Runnels

**Affiliations:** 1 Rutgers-Robert Wood Johnson Medical School, Deptartment of Pharmacology, Piscataway, 08854 USA; 2 Temple University, Deptartment of Biology, Philadelphia, 19122 USA

## Abstract

In humans, germline mutations in *Trpm6* cause autosomal dominant hypomagnesemia with secondary hypocalcemia disorder. Loss of *Trpm6* in mice also perturbs cellular magnesium homeostasis but additionally results in early embryonic lethality and neural tube closure defects. To define the mechanisms by which TRPM6 influences neural tube closure, we functionally characterized the role of TRPM6 during early embryogenesis in *Xenopus laevis*. The expression of *Xenopus* TRPM6 (XTRPM6) is elevated at the onset of gastrulation and is concentrated in the lateral mesoderm and ectoderm at the neurula stage. Loss of XTRPM6 produced gastrulation and neural tube closure defects. Unlike XTRPM6′s close homologue XTRPM7, whose loss interferes with mediolateral intercalation, depletion of XTRPM6 but not XTRPM7 disrupted radial intercalation cell movements. A zinc-influx assay demonstrated that TRPM6 has the potential to constitute functional channels in the absence of TRPM7. The results of our study indicate that XTRPM6 regulates radial intercalation with little or no contribution from XTRPM7 in the region lateral to the neural plate, whereas XTRPM7 is mainly involved in regulating mediolateral intercalation in the medial region of the neural plate. We conclude that both TRPM6 and TRPM7 channels function cooperatively but have distinct and essential roles during neural tube closure.

## Introduction

TRPM6 is one of two mammalian bifunctional proteins with ion channel and kinase domains; the other is TRPM7, a close homolog. The channels are similarly permeable to a wide range of divalent cations, including Mg^2+^, Ca^2+^, and Zn^2+^ ^1–3^. The TRPM6 ion channel was initially identified as the protein whose gene is mutated in the autosomal recessive disorder familial hypomagnesemia with secondary hypocalcemia (HSH)^4,5^. The HSH disorder is characterized by very low Mg^2+^ and Ca^2+^ serum levels. Shortly after birth affected individuals exhibit neurologic symptoms of hypomagnesemic hypocalcemia, including seizures and muscle spasms. Surprisingly, knockout of TRPM6 in mice was reported to be embryonically lethal^6,7^. In one of these studies, homozygous TRPM6 knockout embryos died by embryonic day 12.5 (E12.5) and exhibited neural tube closure defects^6^. Ten percent of TRPM6-null homozygotes had spina bifida occulta, and thirty percent exhibited exencephaly^6^. The expression pattern of TRPM6 during mouse embryogenesis further demonstrated a significant increase in TRPM6 RNA expression at E10 through E15 coincident with the timing of neural tube closure. Surviving heterozygous TRPM6 knockout mice exhibited mild hypomagnesemia, consistent with TRPM6′s reported role in magnesium homeostasis^6^. Interestingly, dams fed with a high Mg^2+^ diet slightly suppressed the embryonic lethality caused by knockout of TRPM6, suggesting that embryonic lethality may be partially due to a Mg^2+^ deficiency in the developing embryo^6^. In a more recent study by Chubanov and colleagues, a mouse strain carrying a gene-trap mutation in Trpm6 (Trpm6^βgeo^) was used to demonstrate that Trpm6^βgeo/βgeo^ embryos survive until E10.5, with no individual embryos surviving past E14.5^8^. The investigators performed *in situ* analysis and did not observe TRPM6 expression in the neural tube but instead detected expression of the channel in syncytiotrophoblast cells within the placental labyrinth. Inductive coupled plasma mass spectrometry analysis of whole E9.5 Trpm6^βgeo/βgeo^ embryos found a decrease in Mg^2+^ content compared to control embryos. To determine whether TRPM6 activity in extra-embryonic cells underlies the lethality of Trpm6 null embryos, the investigators crossed a mouse strain with a ‘floxed’ (Trpm6^fl^) allele with Sox2-Cre transgenic mice, which drives recombination in cells from the epiblast but not as efficiently in extra-embryonic tissues^9,10^. Trpm6^Δ17/Δ17^;Sox2-Cre pups were born at the expected Mendelian ratio, suggesting that the embryonic mortality of Trpm6-deficient mice was mainly due to the loss of TRPM6 activity in placental cells.

By comparison, mouse embryo expression of TRPM7 is dramatically increased from E10.5 to E11.5, and global expression is observed through E14.5^11^. Jin and colleagues generated conditional knockout mice of TRPM7 using a tamoxifen-inducible and multiple tissue-specific Cre recombinase lines^12^. Tamoxifen-dependent deletion of TRPM7 at E7.5 to E8.5 caused embryonic lethality within 48-72 hours, while the depletion at E14.5 did not cause embryonic lethality, with mutant mice developing normally. Beyond TRPM7′s function in early embryogenesis, the channel also has broad roles during organogenesis, including nephrogenesis, cell-cell adhesion during urogenesis, the development of neural-crest-derived pigment cells, and myocardial cell proliferation during early cardiogenesis^12,13^. Additional studies in zebrafish have also highlighted functions for TRPM7 in pigmentation and pancreas development^14,15^. We previously employed the established *Xenopus laevis* model system to conduct a detailed analysis of TRPM7’s function during early embryogenesis. Depletion of TRPM7 from developing *Xenopus laevis* embryos using anti-sense morpholino technology produced defective gastrulation phenotypes, consistent with the phenotype documented in mice^16^. In *Xenopus laevis* TRPM7 (XTRPM7) depleted embryos, axial extension was impaired, resulting in a severe dorsal-flexure and failure of the neural tube to close. Importantly, our studies demonstrated a fundamental role of the channel but not the kinase domain for gastrulation and neural tube closure. As the phenotypes caused by depletion of TRPM7 in *Xenopus* could be suppressed by Mg^2+^ supplementation as well as by expression of the Mg^2+^ transporter SLC41A2, our studies suggested a requirement for Mg^2+^ for gastrulation. The phenotypes caused by depletion of XTRPM7 could also be rescued by expression of exogenous TRPM6, suggesting that TRPM6 could functionally compensate for TRPM7 during early embryogenesis^17,18^. To better understand the role of TRPM6 during embryogenesis, we functionally investigated the role of this protein during *Xenopus* development. Here we report that TRPM6 functions independently of TRPM7 to influence radial cell intercalation, playing a unique but complementary role to TRPM7 for successful completion of gastrulation and neural tube closure.

## Results

Deletion of *TRPM6* in mice results in early embryonic lethality in one study, neural tube closure defects were observed, but whether TRPM6 functions directly or indirectly to influence this process remained unclear. To gain insight into TRPM6’s role during embryogenesis we undertook a study of the channel in *Xenopus laevis*, which is extensively used to study neural tube closure. As a first step to investigate the role of the channel, we first cloned *Xenopus* TRPM6 (XTRPM6) and observed that this protein that shares a sequence identity of 63% with that of the human protein. XTRPM6 domain architecture (Supplementary Figure 1a) is further similar to its vertebrate orthologs, exhibiting high sequence identity and similarity within its channel and kinase domains (Supplementary Figure 1). XTRPM6 shares a sequence identity of 51% with XTRPM7.

**Figure 1:**
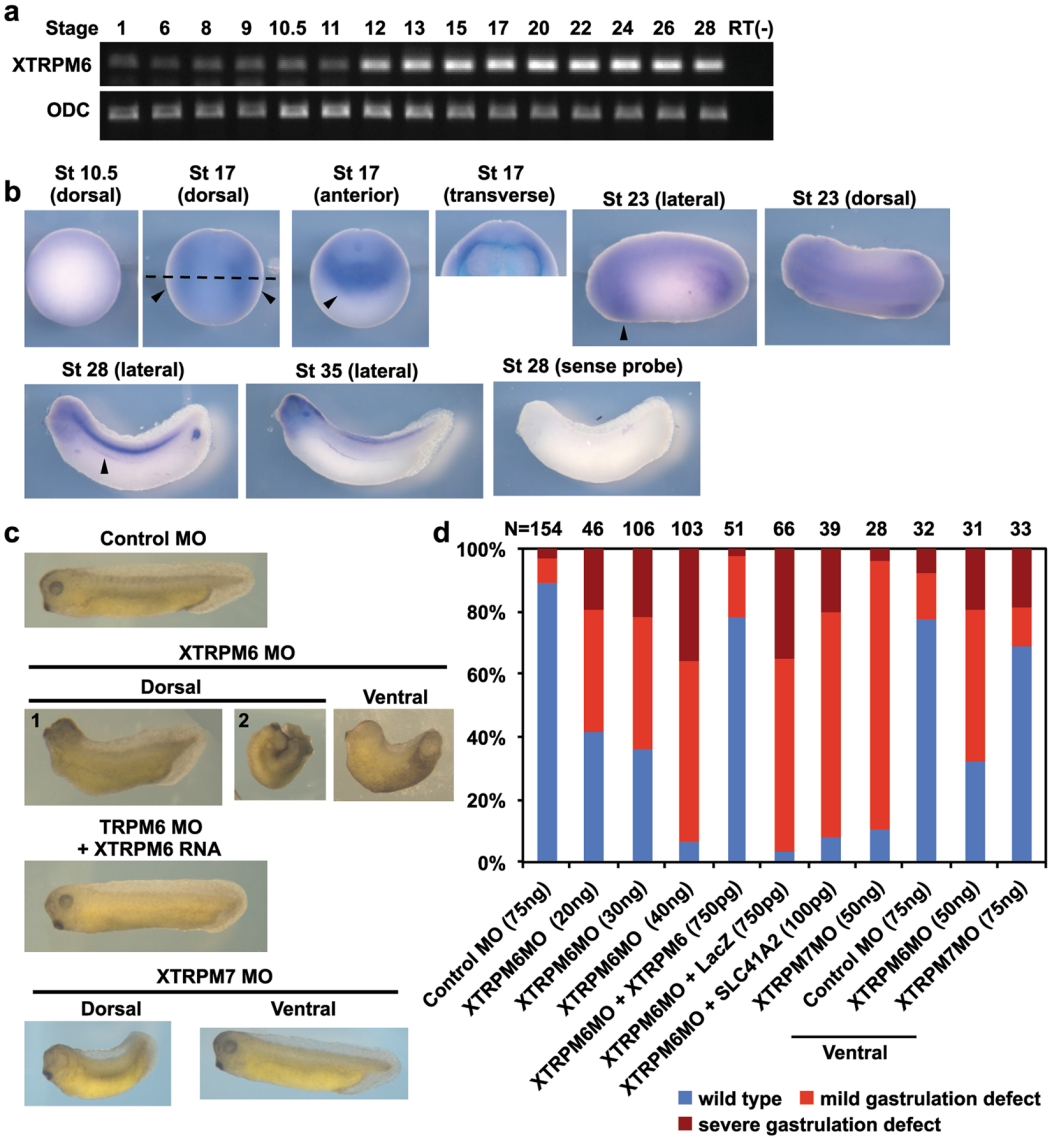
Depletion of XTRPM6 caused gastrulation defects during *Xenopus* embryogenesis. (**a**) Temporal expression pattern of XTRPM6. The expression level of XTRPM6 RNA was assessed by RT-PCR analysis. ODC was used as an internal control. RT(-) is without reverse transcriptase as a negative control. Full gel images shown in Supplementary Figure 5. (**b**) Spatial expression pattern of XTRPM6. Whole-mount *in situ* hybridization was performed using an XTRPM6 anti-sense RNA probe. No signal was detected using an XTRPM6 sense RNA probe. Arrowheads for stage 17 (dorsal) indicate lateral staining; the dotted line indicates where the transverse section was done. Arrowhead for stage 17 (anterior) indicates strong anterior staining overlapping with the cement gland and the pre-placodal region. Arrowheads for stage 23 and stage 28 indicate XTRPM6 expression in primary heart field and pronephric duct, respectively. St: stage. (**c**) TRPM6 is required for gastrulation. A control MO or XTRPM6 MO was injected into the two dorsal or ventral blastomeres at the 4-cell stage, and the phenotype was observed at tadpole stages. Dorsal injection of XTRPM6 MO or XTRPM7 MO caused gastrulation defects. Ventral injection of XTRPM6 MO but not XTRPM7 MO caused a shortened and curved axis. For the rescue experiments, 40 ng of the XTRPM6 MO was co-injected with the specified mRNAs. (**d**) Quantification of phenotypes scored at stage 28–30. The collective total number of injected embryos from all experiments is indicated above each bar.

We next examined the temporal and spatial expression pattern of XTRPM6 by RT-PCR and *in situ* hybridization respectively. RT-PCR analysis revealed a low level of XTRPM6 RNA expression maternally until the early gastrula stage, after which it’s level dramatically increased at late gastrula (stage 12) and was maintained at a high level throughout neurulation (stages 13 to 17) (Fig. 1a). *In situ* hybridization demonstrated broad XTRPM6 RNA expression of TRPM6 at stage 10.5, when gastrulation begins. Unlike XTRPM7, which at the neurula stage (stage 17), shows high expression in the brain and neural plate, XTRPM6 was weakly expressed in the neural plate, and high level of XTRPM6 RNA was detected in the lateral and anterior regions (Fig. 1b, stage 17). Anterior expression of XTRPM6 overlapped with the cement gland and the pre-placodal region. Transverse section of stage 17 embryos demonstrated stronger expression of XTRPM6 in the deep layers of the embryo, with a weaker expression on its surface. At stage 23, following organogenesis, XTRPM6 expression was observed in the notochord, spinal cord, and primary heart field. At tadpole stage (stage 28 and 35), XTRPM6 was highly expressed in the notochord, forebrain, pronephric duct and heart and weakly expressed in the spinal cord (Fig. 1b).

To delineate the role of XTRPM6 in early development, we performed a loss-of-function study by microinjection of an antisense morpholino oligonucleotide (MO) targeting XTRPM6 (XTRPM6 MO) (Supplementary Figure 2a). The XTRPM6 MO efficiently inhibited translation of a Myc-tagged 5′UTR-XTRPM6 reporter construct, but not the HA-tagged XTRPM6 rescue construct, in which the XTRPM6 MO binding site had been mutated to prevent morpholino binding (Supplementary Figure 2b and c). A control MO or the XTRPM6 MO was injected into two dorsal or ventral blastomeres of the 4-cell stage embryo, and XTRPM6 morphants were observed at tadpole stages. Dorsal blastomeres are fated to form the neural tube, the cement gland, eyes, brain and central nervous system. Ventral blastomeres give rise to epidermal cells, blood, mesenchyme and gut cells. Most control MO injected embryos developed normally, with just a few exhibiting gastrulation defects due to non-specific toxicity. By contrast, XTRPM6 MO dorsally injected embryos developed normally through early gastrulation (stages 10–15) but began to produce visible gastrulation defects in a dose-dependent manner at the tadpole stage (Fig. 1c and d). Similar to what we previously observed in XTRPM7-depleted embryos, XTRPM6 MO injected embryos displayed curved and shortened axis (mild gastrulation defect, panel 1). For more severely defected embryos, we observed a very short and curved axis with open neural tube (severe gastrulation defect, panel 2). Some embryos exhibited slightly smaller head structures, including eyes, cement gland, and brain. At later stages XTRPM6 injected embryos exhibited defects in body pigmentation. Importantly, defects induced by XTRPM6 MO were rescued by co-injection of the XTRPM6 MO with HA-XTRPM6 RNA harboring a mutated MO binding site, but not by co-injection of the XTRPM6 MO with LacZ RNA. We also investigated whether the phenotype produced XTRPM6 MO could be rescued by expression of the Mg^2+^ transporter SLC41A2 and observed only a modest reduction in the number of embryos with severe gastrulation defects (Fig. 1d). However, supplementation of the medium with excess Mg^2+^ was more effective in rescuing the gastrulation defects caused by the XTRPM6-MO in a dose-dependent manner (Supplementary Figure 3), suggesting that similar to what we previously reported for the XTRPM7 MO, the gastrulation defects produced by the XTRPM6 MO is dependent, at least in part, on Mg^2+^ ^16^. Surprisingly, we observed that ventral injection of XTRPM6 MO, but not XTRPM7 MO, resulted in a curved and shortened axis. These results demonstrated that XTRPM6 is required for gastrulation during *Xenopus* embryogenesis, but suggested that the respective roles of the two channels may be different.

We first asked whether the defect in neural tube closure in XTRPM6-depleted embryos could have been caused by a defect in mesodermal specification. We examined the expression of mesodermal marker genes by RT-PCR. The XTRPM6 MO was injected into the dorsal marginal zone at the 4-cell stage, and RNA was extracted at stage 13. The expression levels of the dorsal mesodermal markers Goosecoid, Siamois, Noggin, Chordin, the ventral mesodermal marker XVent, and the pan-mesodermal marker Xbra were not significantly changed in either XTRPM6 MO or XTRPM7 MO morphants (Supplementary Figure 4). This result indicates XTRPM6 does not contribute to mesodermal specification, indicating that XTRPM6 is affecting neural tube closure by another mechanism.

### XTRPM6 is required for neural tube closure

**Figure 2:**
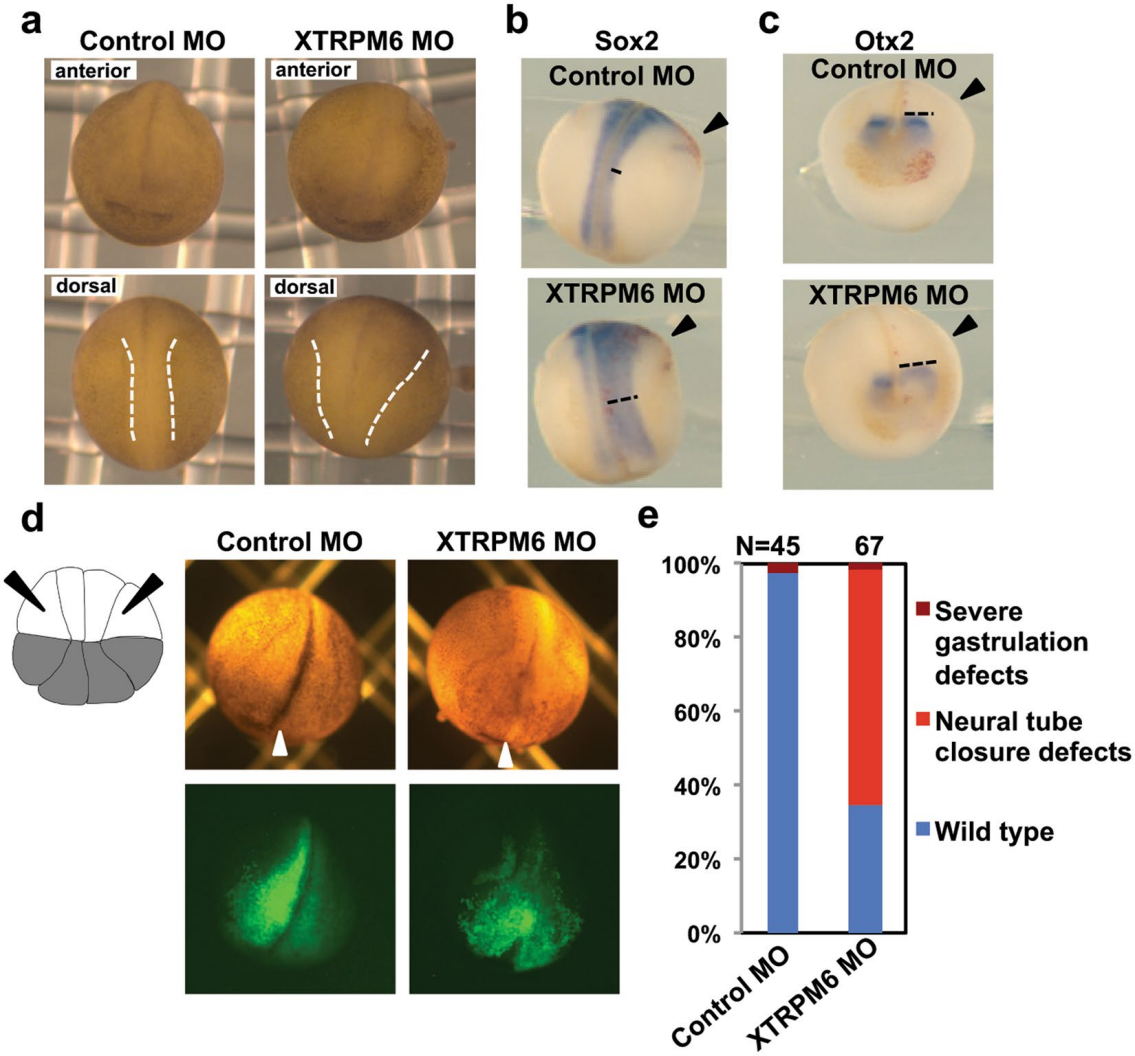
XTRPM6 is required for neural tube closure. (**a**) A control MO or XTRPM6 MO was injected into the two dorsal blastomeres at the 4-cell stage embryos. At stage 20, depletion of XTRPM6 caused neural tube closure defect. Dotted lines indicate the neural fold. (**b**) and (**c**) Visualization of the neural plate by staining with Sox2 (b) and Otx2 (c). The *in situ* hybridization of Sox2 (pan-neural marker) and Otx2 (pre-chordal neural plate marker) indicate that a wider neural plate is observed on the XTRPM6 MO injected side compared to that of uninjected side (black dotted lines). (**d**) The XTRPM6 MO or a control MO was co-injected with GFP RNA into the animal lateral blastomeres of the 16-cell embryo to target the lateral neural plate and epidermal tissue, where XTRPM6 is highly expressed. The lateral animal blastomeres are indicated with black arrows in the illustration of the 16-cell embryo. White arrowheads indicate midline of the embryo. (**e**) Quantification of the results from (**d**). The collective total number of injected embryos from all experiments is indicated above each bar.

To further investigate the function of XTRPM6 during embryogenesis, we carefully observed XTRPM6 morphants during neurulation. During neurulation, several morphogenetic events, including neural fold elevation, convergent extension, and neural plate bending occur in neuroectoderm and mesoderm. At stage 20, when the neural tube has closed, XTRPM6 MO injected embryos exhibited a wider neural plate, and the neural fold did not elevate as well as in embryos injected with the control MO (Fig. 2a, dotted lines). To visualize the neural plate clearly, we performed *in situ* hybridization with Sox2 (a pan-neural marker) and Otx2 (anterior neural plate marker). Both Sox2 and Otx2 staining were wider on the side of the embryo injected with the XTRPM6 MO (arrowheads) compared to the embryo’s uninjected side (Fig. 2b and c, dotted lines). Since XTRPM6 is expressed lateral to the neural plate, we depleted XTRPM6 specifically in lateral tissue by injecting the XTRPM6 MO into the lateral animal blastomeres of the 16-cell stage embryo and observed the impact on neural tube formation. GFP RNA was co-injected with the XTRPM6 MO to track the injected cells. Injection of XTRPM6 MO laterally produced neural tube closure defects, in which the neural folds were not elevated, and the border of the neural plate was not clear (Fig. 2d and e). By comparison, lateral injection of the control MO did not produce any visible defects in the embryos. The GFP distribution in the XTRPM6 MO-injected embryo was wider than that of the control MO-injected embryo, indicating a failure of the neural fold to move to the midline. These experiments indicate a role for XTRPM6 in regulating cell movements during neural tube closure lateral to the neural plate.

### XTRPM6 is not required for mediolateral intercalation

**Figure 3:**
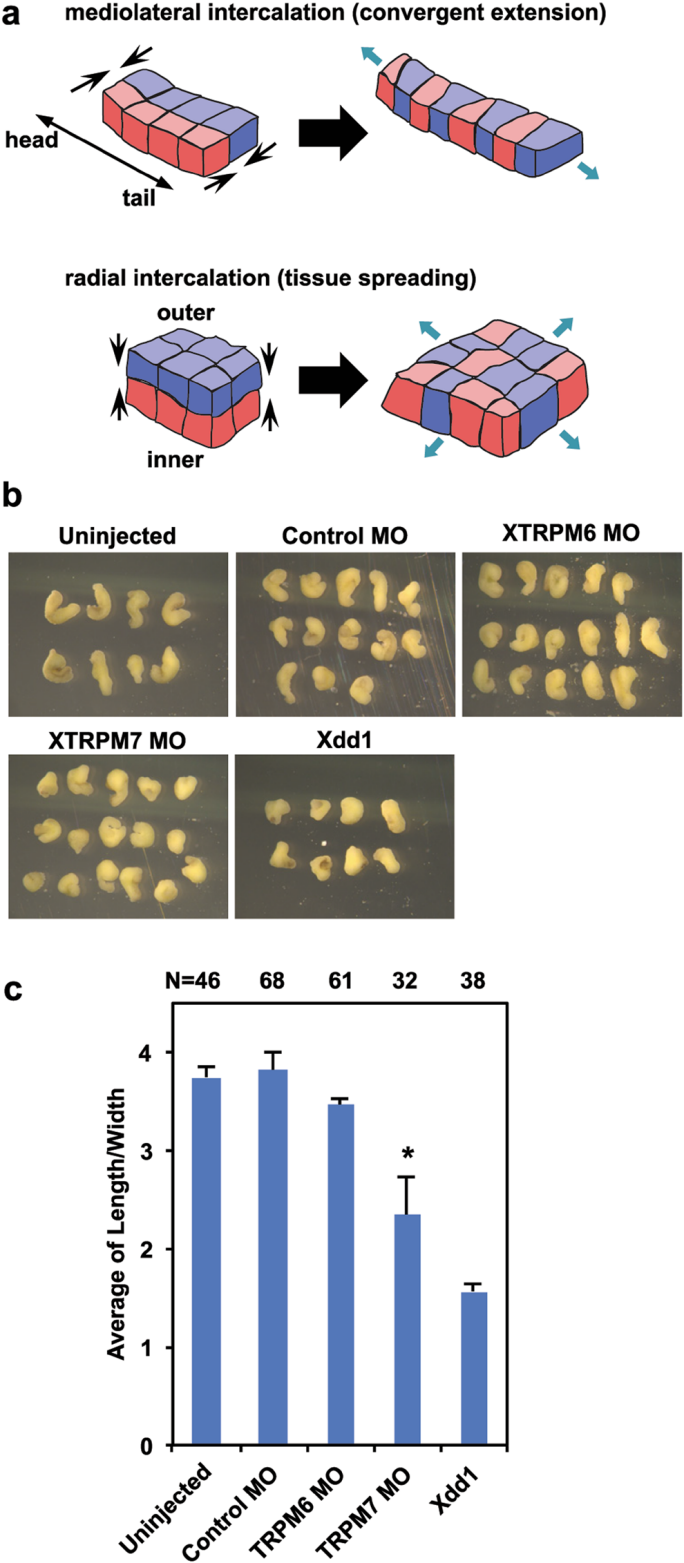
TRPM7 but not TRPM6 is required for mediolateral intercalation. (**a**) Schematic illustration of mediolateral and radial intercalation. During gastrulation and neurulation, mesodermal and ectodermal cells move and exchange their positions with one another, which is referred to as cell intercalation. Depending on the direction of the cell movement, intercalation can be divided into mediolateral (upper illustration) and radial (lower illustration) intercalation. Black arrows indicate the direction of cell movement. Mediolateral intercalation results in tissue lengthening and narrowing, whereas radial intercalation results in tissue spreading as the number of layers are reduced (indicated solid green arrows). (**b**) A Keller explant assay was performed to investigate XTRPM6’s effect on mediolateral intercalation. The designated morpholinos or dominant negative Dishevelled (Xdd1) RNA were injected into two dorsal blastomeres of 4-cell stage embryos. The dorsal marginal zone, which undergoes mediolateral intercalation, was dissected at stage 10.5. Embryos injected with Xdd1, and the TRPM7 MO were used as positive controls for their inhibition of mediolateral intercalation. (**c**) Quantification of the results from Keller explant assay. Explants were scored by the ratio of length/width. The collective total number of injected embryos from all experiments is indicated above each bar. Error bars indicate standard error. *P = 0.017.

During neurulation, the neural ectoderm becomes thickened and forms the neural plate, which afterward bends to form neural folds that subsequently move towards the midline to close the neural tube. Several types of cell migration are required to accomplish neural tube formation. Cell intercalation is one of these cell movements, in which cells exchange their positions between neighboring cells. Depending on the direction, intercalation can be divided into two types of cell movements: mediolateral and radial intercalation (Fig. 3a). During mediolateral intercalation, polarized cells in the same plane intercalate with each other to lengthen the embryo in the head-tail direction (extension), which narrows the embryo (convergence) along the orthogonal axis (Fig. 3a upper illustration). Mediolateral intercalation is also referred to as convergent extension, which occurs in *Xenopus* during gastrulation in the dorsal marginal zone and the neural plate during neural tube closure. It is well known that the non-canonical Wnt pathway operating through Dishevelled-dependent activation of Rho and Rac GTPases plays a pivotal role in controlling convergent extension^19^. During radial intercalation, cells in multiple layers exchange their positions, resulting in the thinning of tissue and the spreading of cells from inside to outside of the embryo (Fig. 3a lower illustration). Radial intercalation in *Xenopus* embryogenesis is observed in animal pole cells during the blastula stages and in the ventral marginal zone and lateral plate during gastrulation and neurulation. Importantly, a failure of either type of cell intercalation can produce a range of neural tube closure defects, including spina bifida, one of the most common human birth defects.

To investigate whether XTRPM6 has any role in the cell intercalation, we first performed a Keller explant assay to examine XTRPM6 potential contribution to mediolateral intercalation. In the Keller explant assay, the dorsal lip region of stage 10.5 embryos are dissected and cultured and allowed to undergo mediolateral intercalation until the non-dissected embryos reach stage 20–22. We previously demonstrated that depletion of XTRPM7 strongly inhibits the elongation of explants and disrupts cell polarization^16^. Injection of our positive controls, the XTRPM7 MO or a dominant-negative form of Dishevelled lacking the PDZ domain (Xdd1), strongly inhibited elongation of the dissected explants (Fig. 3b and c). By contrast, XTRPM6 MO injected explants were well elongated, and the length/width ratio of TRPM6 MO injected explants were not significantly different from uninjected explants or explants derived from embryos injected with the control MO (Fig. 3b and c). These data confirm a role for XTRPM7 in mediolateral intercalation but also demonstrates that XTRPM6 does not contribute to this process, indicating that XTRPM6 affects neural tube closure through a distinct mechanism.

### XTRPM6 is required for radial intercalation during early neurulation

**Figure 4:**
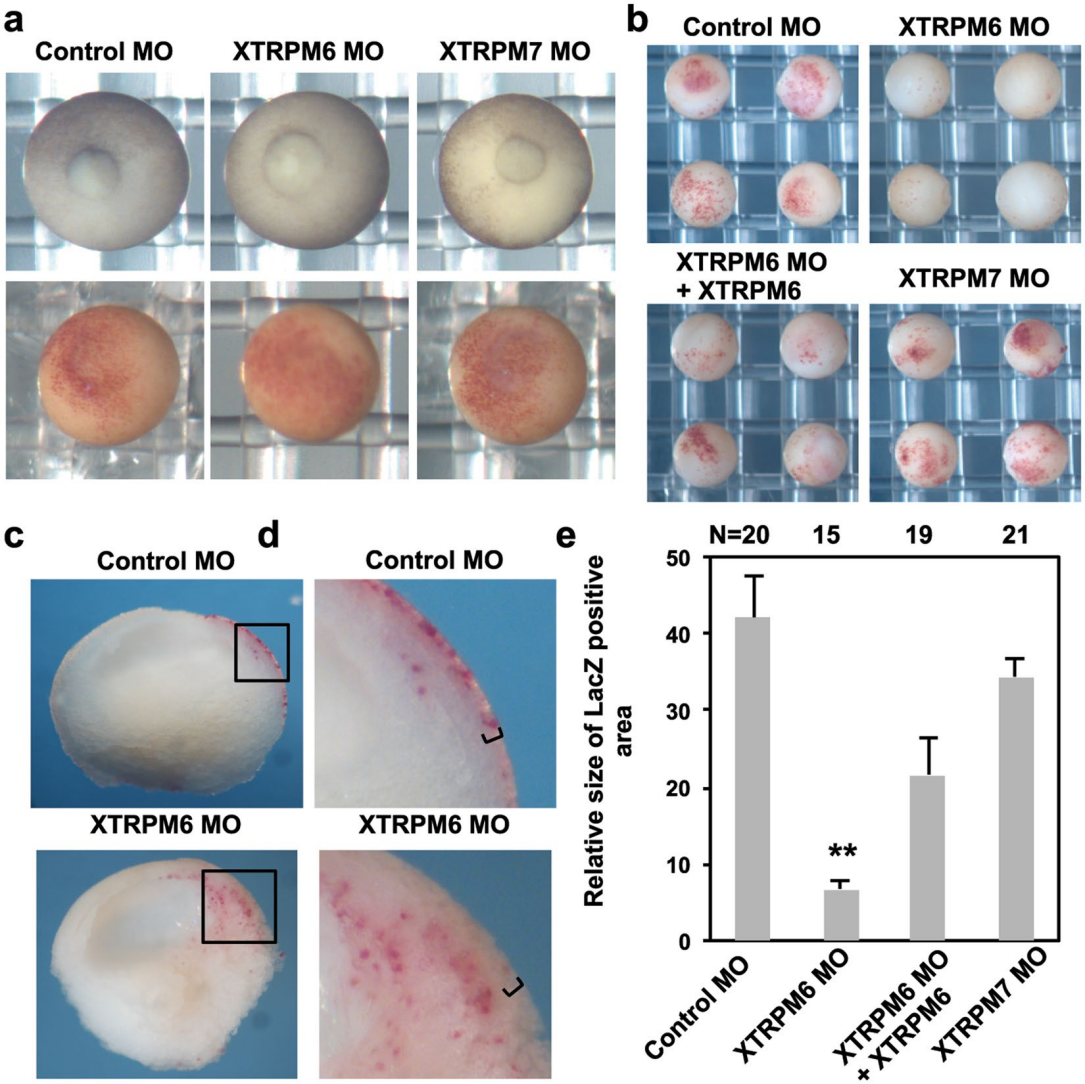
XTRPM6 but not XTRPM7 is required for radial intercalation. (**a**) Control and XTRPM6 MOs were injected into the animal pole at 2-cell stage embryo. LacZ RNA was co-injected with MO as a tracer. At late gastrula stage (stage 12-13) the process of blastopore closure was observed (upper panels). Embryos were stained for β-galactosidase activity using Red-gal as a substrate (lower panels) to visualize the distribution of LacZ-positive cells. No defect in blastopore closure was observed in XTRPM6 MO-injected embryos. (**b**) Control and XTRPM6 MOs were laterally injected into two ventral blastomeres with LacZ RNA at 4-cell stage embryo. Embryos were stained for β-galactosidase activity at stage 14–15. XTRPM6 MO injected embryos had very little staining on the embryo surface compared to control MO-injected embryos. (**c**) and (**d**) Red-gal stained embryos injected with the XTRPM6 MO or control MO were partially dissected. For control MO-dissected embryos, strong staining was observed in the outer epithelial ectoderm layer of the embryo (indicated with brackets). In contrast, strong staining was not readily observed at the outer epithelial ectoderm layer (indicated with brackets) for TRPM6 MO-dissected embryos; instead strong staining was observed in deeper layers of the embryo and on the archenteron roof. (**e**) Quantification of surface staining from (**b**). The collective total number of injected embryos from all experiments is shown above each bar. Error bars indicate standard error. **P < 0.01.

Our experiments indicate that XTRPM6 does not significantly affect mediolateral intercalation, which led us to investigate the channel’s impact on radial intercalation. As mentioned previously, during embryogenesis radial intercalation is first observed in animal pole cells during the blastula stages. A failure of the animal pole cells to radially intercalate will lead to a defect in blastopore closure. To investigate the involvement of XTRPM6 in the radial intercalation of animal pole cells we injected the XTRPM6 MO with LacZ RNA as a tracer into the animal pole at the 2-cell stage. Neither injection of the XTRPM6 MO nor the XTRPM7 MO produced any defects in blastopore closure (Fig. 4a upper panels). We also observed that there was no significant difference in the distribution of LacZ-positive cells among all injected embryos (Fig. 4a lower panels), which also indicates that radial intercalation of the animal pole cells had not been disrupted.

During early neurulation, while mediolateral intercalation occurs near the midline of the embryo, radial intercalation takes place in the lateral plate and ventral marginal zone^20^. Thus a failure of radial cell intercalation at this stage, which is controlled by complex signaling pathway distinct from non-canonical Wnt signaling, can also lead to neural tube closure defects. Our *in situ* expression data indicate that XTRPM6 is expressed laterally to the neural plate at the neurula stage (Fig. 1b); whereas we found previously XTRPM7 to be expressed primarily in the neural plate^16^.

In late gastrulation and early neurulation, lateral mesoderm and the ventral marginal zone undergo radial intercalation. To investigate the specific contribution of XTRPM6 and XTRPM7 to radial intercalation at these stages, we laterally injected XTRPM6 and XTRPM7 MOs with LacZ RNA as a tracer into ventral blastomeres of 4 cell embryos, which allowed us to target the tissue undergoing radial intercalation during neural tube closure. Injected embryos were stained for β-galactosidase activity at the early neurula stage (stage 14–15). Control MO injected embryos showed a spread distribution of LacZ positive cells on the surface of the embryo, which indicated proper intercalation of the deep lateral cells. By contrast, very few LacZ positive cells were detected on the surface of XTRPM6 MO injected embryos (Fig. 4b and e). Partial dissection of stained embryos revealed that most of the LacZ-positive cells were observed on surface layers in control MO-injected embryos, whereas a majority of LacZ-positive cells were found in deep layers and on the archenteron roof of XTRPM6 MO-injected embryo (Fig. 4c and d). We observed no obvious difference in the overall number of stained cells between control MO- and XTRPM6-injected embryos. Rather, 10% of staining in Control MO-dissected was found interior, whereas, for XTRPM6 MO-dissected embryos, 75% was found interior. Thus the above data is consistent with a failure of radial intercalation (Fig. 4e). The defect of radial intercalation by XTRPM6 MO was partially rescued by co-injection of XTRPM6 mRNA with the XTRPM6 MO. In contrast to the staining defects produced by the XTRPM6 MO, XTRPM7 MO injected embryos showed only a modest reduction in surface LacZ staining. These observations indicate that XTRPM6, but not XTRPM7, is selectively involved in the radial intercalation during early neurulation. The different effects between TRPM6 and TRPM7 on cell intercalation also suggest that both channels appear to have separate yet complementary functions for the successful completion of neural tube closure.

### XTRPM6 does not require XTRPM7 to mediate divalent-cation influx

**Figure 5:**
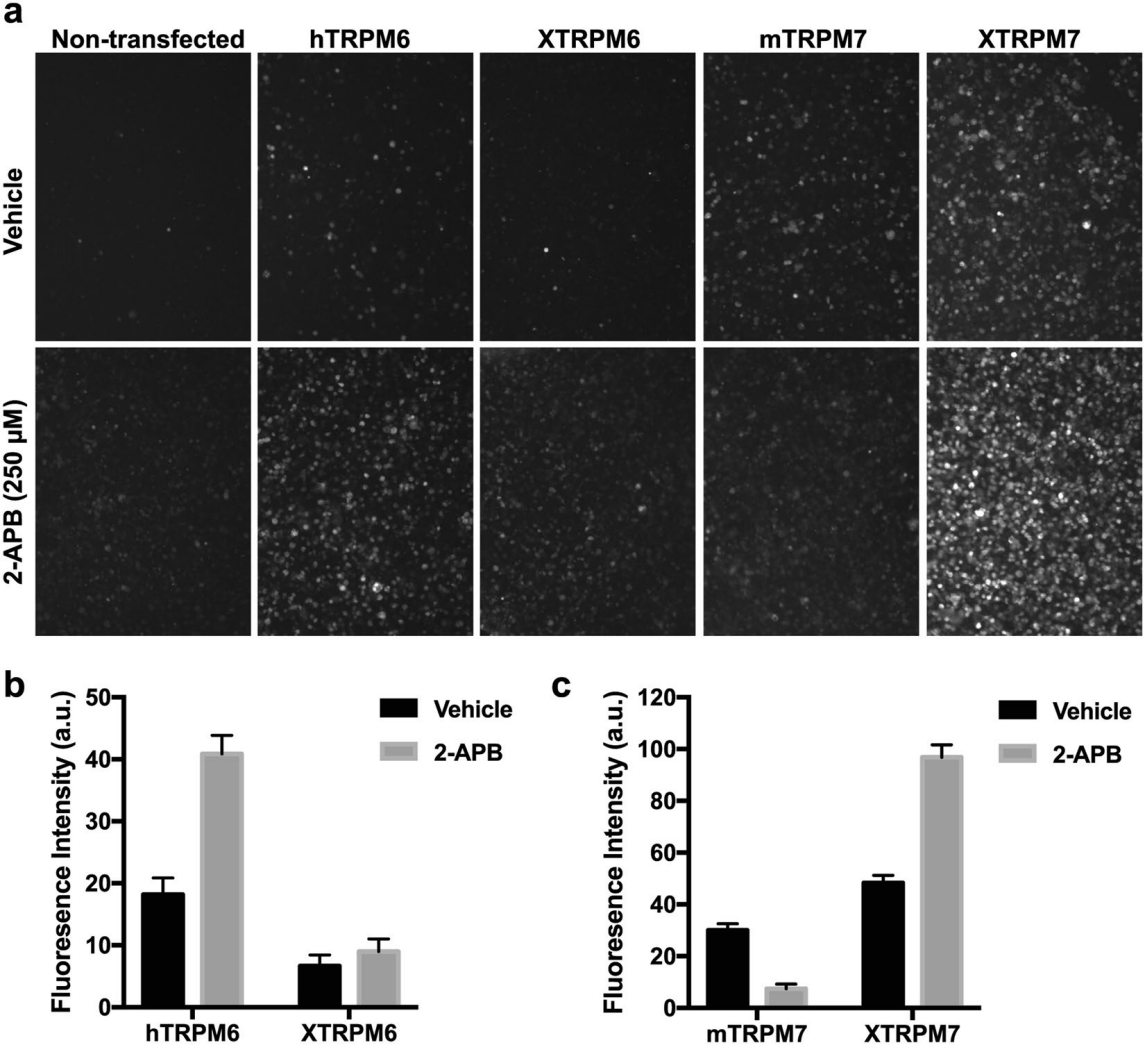
TRPM6 mediates divalent-cation influx. (**a**) Zinc-influx assay was employed to assess the channel function of XTRPM6, hTRPM6, mTRPM7, and XTRPM7 in intact cells. hTRPM6 and mTRPM7 were used as positive controls. Uptake of Zn^2+^ could be detected in TRPM7^−/−^ HEK293T cells transiently transfected with XTRPM6, XTRPM7, mTRPM7, and hTRPM6. Zn^2+^-uptake by XTRPM6, hTRPM6, and XTRPM7 could be pharmacologically enhanced by application of 250 μM 2-APB, whereas Zn^2+^-uptake by mTRPM7 was suppressed by application of 250 μM 2-APB. (**b** and **c**) Quantification of fluorescence intensity expressed in arbitrary units (a.u.) from the results shown in (**a**).

Our studies indicate that XTRPM6 operates independently of XTRPM7 in the lateral mesoderm to accomplish radial intercalation during neural tube closure, yet whether TRPM6 is functional in the absence TRPM7 has been controversial. In previous studies, TRPM6 was reported to be dependent upon TRPM7 for cell surface expression^17,18^. Full-length variants of TRPM6 failed to form functional channel complexes when heterologously expressed in HEK-293 cells and *Xenopus* oocytes^17^. By contrast, Voets and colleagues were able to measure functional TRPM6 channels when the protein was heterologously expressed in HEK-293 cells^2^. As did also Yue and colleagues, who made single channel recordings to differentiate between TRPM7 and TRPM6 single channel conductance^3^. More recently, Ferioli and colleagues showed that expression of TRPM6 in *Trpm7*-deficient trophoblast stem cells yields TRPM6 currents, adding to the evidence that TRPM6 can form independent channels^21^. To assess the potential of TRPM6 to form functional channels in intact cells in the absence of TRPM7 we expressed XTRPM6 and hTRPM6 in TRPM7-knockout HEK293T cells (TRPM7^−/−^ HEK293T) and employed a zinc-influx assay similar to the one used previously to follow Zn^2+^-influx by TRPM7^22^. In addition to Mg^2+^ and Ca^2+^, TRPM7 and TRPM6 are highly permeable to Zn^2+^ ^1–3^. In this assay, the external buffer is supplemented with 30 μM ZnCl_2_ so that Zn^2+^ can be used as a tracer to follow divalent cation uptake through the channels. Transient expression of human TRPM6 (hTRPM6) or XTRPM6 in TRPM7^−/−^ HEK293T cells increased Zn^2+^-uptake compared to non-transfected cells (Fig. 5a and b), confirming that TRPM6 can form functional channels on its own. We further characterized the channels by assessing the effect of 2-APB on zinc-uptake. The channel activity of hTRPM6 has been shown to be pharmacologically activated by 250 μM 2-APB, whereas mTRPM7 is inhibited by 2-APB at concentrations less than 1 mM^3^. As expected, we observed an increase in Zn^2+^-uptake of cells transiently expressing hTRPM6 and treated with 250 μM 2-APB (Fig. 5a and b)^3^. As previously reported, we also observed that application of 250 μM 2-APB inhibited Zn^2+^-uptake in mouse TRPM7 (mTRPM7) expressing cells, whereas for XTRPM7 application of 250 μM 2-APB stimulated XTRPM7 channel function (Fig. 5a and c)^3^. Also, cells that were transfected with XTRPM6 showed a more modest increase in Zn^2+^-uptake in response to 2-APB compared to that observed for hTRPM6, indicating that the sensitivity of TRPM6 and TRPM7 to 2-APB is not strictly conserved among species. Indeed, zebrafish TRPM7 channel function was also recently shown to be potentiated by 2-APB^23^. Nevertheless, the results of our Zn^2+^-uptake assay indicate that TRPM6 can form functional channels independent of TRPM7, which is supportive of a model in which XTRPM6 but not XTRPM7 is selectively required for radial intercalation during neural fold closure.

## Discussion

Our studies have uncovered a unique role for XTRPM6 in regulating radial cell intercalation during neural tube closure. The temporal and spatial expression pattern of XTRPM6 is further distinct from that of TRPM7 during early embryogenesis. Whereas expression of XTRPM7 is maternal and temporally uniform during embryogenesis, the expression of XTRPM6 is upregulated during the late gastrula stage and remains high throughout neurulation. Spatially, we observed broad XTRPM6 RNA expression at stage 10.5, a time when gastrulation begins. At the neurula stage, XTRPM6 was weakly expressed in the neural plate but is highly expressed in the lateral and anterior regions of the embryo. By contrast, XTRPM7 expression at the neurula stage was elevated in the brain and neural plate, pointing to distinct and non-redundant functions for the two channels during gastrulation and neural tube closure.

**Figure 6:**
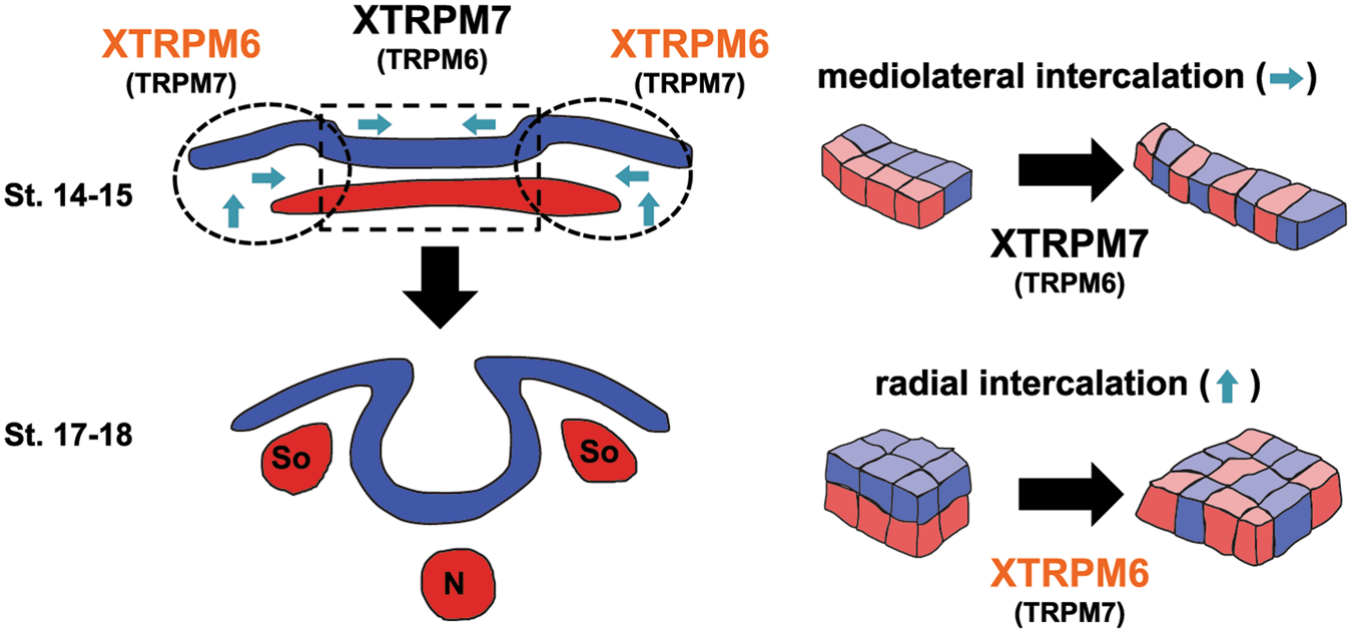
Model of XTRPM6 and XTRPM7 functions during neural tube closure. A transverse section of the neural tissue is shown at the indicated stages (left). The blue tissue represents ectoderm, and the red issue represents mesoderm. “So” indicates Somites. “N” indicates notochord. At the early neurula stage (stage 14–15), XTRPM6 is primarily expressed in the lateral regions (dotted circles) of the embryo, whereas XTRPM7 is mainly found in the medial region (dotted rectangle) of the embryo. Right illustrations show cell movements during mediolateral and radial intercalation. The results of our experiments indicate that XTRPM6 regulates radial intercalation in the lateral region with little or no contribution from XTRPM7 to support neural fold elevation and neuroectodermal cell movement towards the midline. While this radial intercalation in the lateral region is occurring, XTRPM7 is mainly involved in regulating mediolateral intercalation in the medial region of neural plate to contribute to neural tube closure. Our Keller explant data indicate the XTRPM6 may also contribute to a small degree to mediolateral intercalation. Parentheses around TRPM7 and TRPM6 indicate where the channels may have a minor functional role. The above model is based on the present data and previously published results (ref.^16^).

Gastrulation is a complicated morphological process in which multiple deep layers of mesoderm and neural tissue located outside the midline and lateral to the neural plate intercalate, drive the movement of non-neural mesodermal and ectodermal cells for neural fold apposition and subsequent neural tube closure. Our studies indicate that XTRPM7 is required for mediolateral intercalation, which occurs within the midline of the embryo, whereas XTRPM6 is needed for radial intercalation at the lateral plate within non-neural mesoderm (Fig. 6). How XTRPM6 influences radial intercalation requires additional study. Interestingly the difference in the expression pattern of TRPM7 and TRPM6 could explain why the loss of either channel in *Xenopus* or mice produced similarly lethal phenotypes since the channels are both required for different aspects of neural tube closure. However, because the channels are selectively spatially expressed in different tissues, functional compensation cannot occur. Moreover, the expression of XTRPM6 within the non-neural mesoderm and ectoderm could offer a possible explanation for why global knockout of TRPM6 in mice produced severe neural tube defects, as reported by Walder and colleagues, whereas no such phenotypes were reported by Chubanov and colleagues for Trpm6^Δ17/Δ17^;Sox2-Cre mice, in which Cre recombinase expression is driven by the Sox2 promoter. Sox2-Cre mice are commonly employed to drive recombination within the epiblast as compared to extra-embryonic tissues^9,10^. During mouse embryogenesis, expression of Sox2 RNA is observed throughout the epiblast but by mid-late-streak stages, it becomes restricted to presumptive neuroectoderm in the anterior^24^. At the onset of neural plate formation, Sox2 RNA expression is observed in the region of anterior ectoderm, which gives rise to neuroectoderm and anterior surface ectoderm^25^. *In situ* analysis of *Xenopus laevis* embryos demonstrate XTRPM6 RNA expression lateral to the neural plate, whereas Sox2 is found expressed in the neural plate (e.g., Figs 1b and 2b). It’s possible that the absence of neural tube defects in Trpm6^Δ17/Δ17^;Sox2-Cre mice may be due to inefficient disruption of TRPM6 expression in non-neuronal tissues lateral to the neural plate. Alternatively, we cannot rule out the possibility of species-specific differences in which TRPM6 may not play a direct role in neural tube closure in higher vertebrates. Nevertheless, the results obtained by Chubanov and colleagues do point to a critical role for TRPM6 in placental Mg^2+^ transport, which underscores an essential requirement for Mg^2+^ for early stages of development.

There has been controversy as to whether XTRPM6 functions independently of XTRPM7. Our experimental results from *Xenopus* demonstrate that XTRPM7 does not significantly contribute to radial intercalation but that XTRPM6 functions to regulate this process. Moreover, using a Zn^2+^-influx assay, we were able to demonstrate that both hTRPM6 and XTRPM6 are capable of mediating divalent-cation influx when individually expressed TRPM7^−/−^ HEK293T cells^26^. We propose that some physiological processes may only require XTRPM6, but that others may utilize both channels depending on the physiological and developmental setting, as was so elegantly shown by Ferioli and colleagues recently^21^.

Although both TRPM6 and TRPM7 are relatively non-selective divalent channels permeable to Mg^2+^, Ca^2+^, and Zn^2+^, our studies and now others point to a critical role for Mg^2+^ during gastrulation and neural tube closure^16,27^. In our earlier investigation of XTRPM7 in *Xenopus*, we discovered that the gastrulation defects produced by the depletion of XTRPM7 could be rescued by Mg^2+^ supplementation or by expression of the Mg^2+^ transporter SLC41A2^16^. We also found that in TRPM7-knockdown fibroblasts, disruption of polarized cell movements can also be rescued by expression of SLC41A2^28^. Likewise, Mg^2+^ and expression of SLC41A2 could also suppress the gastrulation defects produced by the embryos injected with the XTRPM6 MO. Recently, a polymorphism in human *TRPM6* that correlated with lower serum magnesium was identified in children with meningomyelocele (MMC), a form of spina bifida or neural tube defect in which the spinal canal and the backbone don’t close before birth^29^. Thus, evidence for a critical role for Mg^2+^ in neural tube closure continues to accumulate. How Mg^2+^ specifically influences the behavior of motile cells remains poorly understood, and additional experiments are now required to address this critical question.

## Methods

### Reagents

All reagents, unless otherwise stated, were from Sigma-Aldrich (St. Louis, MO).

### Plasmids and oligonucleotides

XTRPM6 cDNA (Genbank Accession # KY849930) was amplified from *Xenopus laevis* pronephros by RT-PCR and first subcloned into pENTR-D (Thermo Fisher Scientific, Waltham, MA) using the following primers (XTRPM6-FOR; 5′-CAC CAT GAA AGT CCA GCT AAA GTC TCA GAA ATC ATG G-3′ & XTRPM6-REV; 5′-TGT CAT ATA GTC AGA CTG GGT GCA ATA CTT ATT TTA TGT GAC AGA A). To make a MO-resistant expression vector, we introduced silent mutations into the XTRPM6 MO binding site and simultaneously added a HA tag we first amplified a small fragment of DNA using the following primers (XTRPM6HAFOR; 5′-CAC CGC GGC CGC ATG TAC CCA TAC GAT GTT CCA GAT TAC GCT AAG GTG CAA TTG AAA TCT CAG AAA TCA TGG ATT GAA GGA ATC TAC AAT AA-3′; XTRPM6-ScaIBluntRev; 5′-GAA ACT GCT AAT TGT AGG CAT GTT GAA TTA CTC GAG-3′). This fragment was cloned into pENTR-D and then a NotI-ScaI fragment was swapped into the original XTRPM6 clone. HA-XTRPM6 was subsequently introduced into pcDNA3.1D/V5-His-TOPO by PCR using XTRPM6HAFOR and XTRPM6REV primers. For *in situ* analysis of XTRPM6 we subcloned a 666 base pair fragment overlapping the 5′ untranslated region of XTRPM6 and part of the coding region by PCR using the following primers: XTRPM6PROBEFOR; 5′-CAC CCA CAG CTG ACC TGG AAG ACA AGT CTT AC-3′, XTRPM6PROBEREV; 5′-GCT TTC AAA GCA TCG GCC ACG TG-3′. The PCR fragment was then subcloned into the pCRII vector. To create Myc-tagged-5-‘UTR-TRPM6 a 369 bp fragment containing the XTRPM6 MO binding site and an NH2-terminal fragment of XTRPM6 was subcloned in frame into the XhoI and XbaI sites of pCS2-MT using the following primers: MYC-5′UTRM6FOR; 5′-CAC CAT CAT CTC GAG CCC CCT GCA AAC ATG AAA GTC CAG CTA AAG-3′ and MYC-5′UTRM6REV; 5′-CAT CAT TCT AGA CTA CAG ATC CAA CTC GGT ATC ATA GGA TAT CCT TAT G-3′. XTRPM7 (Genbank Accession #: GQ304750.1) was similarly cloned by RT-PCR using the following primers: (XTRPM7FOR; 5′-CAC CTG CTT GTG TCT GCA CGT TGA AGA GAA TGT CCC AGA AAT CTT GGA TAG-3; XTRPM7REV; 5′-TTA CAA CAT GAG ACG GAT AGA GTT TTT CGA TTC TTT GGT GAA ATG ATC AGG ATC TG-3′, XTRPM7FLAGFOR; 5- CAC CAT GGA CTA CAA GGA CGA CGA TGA CAA GTC CCA GAA ATC TTG GAT AGA AAC TTC TTT CAC CAG AAG GGA ATG TAC CTA C-3′). The DNA for XTRPM6 and XTRPM7 can only be propagated and amplified in CopyCutter EPI400 *E.Coli* cells (Epicentre, Madison, WI) because both XTRPM6 and XTRPM7 DNA are either toxic to *E.Coli* or are unstable.

### Cell lines

The 293 T cell line was purchased from ATCC^®^ (CRL-3216). The cells were maintained in a Dulbecco’s Modified Eagle Medium (DMEM), high glucose media with 10% FBS in a humidified 37 °C, 5% CO_2_ incubator. TRPM7^−/−^ HEK293T were obtained from Dr. David Clapham (Janelia Research Campus, HHMI, Ashburn, VA) and were maintained as previously described^26^.

### Embryo manipulations

Embryo manipulations were performed as previously described^30,31^. Embryo injections were performed using *in vitro* transcribed RNAs or MOs. For the radial intercalation assay during early gastrulation, the MOs and LacZ RNA were co-injected into animal pole of 2-cell embryos. The injected embryos were then fixed at stage 12–13 and subjected to β-galactosidase staining using Red-Gal. For the radial intercalation assay during neurulation, the MOs and LacZ RNA were laterally co-injected into the two ventral blastomeres of 4-cell stage embryos. The injected embryos were then fixed at stage 14–15 and subjected to β-galactosidase staining. The *Xenopus laevis* XTRPM6 morpholino (XTRPM6 MO) was 5-TTAGCTGGACTTTCATGTTTGCAGG-3 and was synthesized by Gene-Tools (Philomath, OR). The XTRPM7 MO (XTRPM7 MO3) was 5′-GGGACATTCTGTTCAACGTGCAGAC-3′. The control MO was purchased from Gene-Tools. All experimental animal protocols were approved by the Rutgers-RWJMS Institutional Animal Care and Use Committee committee and conducted in accordance with the relevant guidelines and regulations.

### Detection of protein expression

The Anti-c-Myc antibody was used to detect Myc-tagged-5′UTR-TRPM6 from *Xenopus* embryos. The sheep anti-mouse secondary antibody was purchased from GE Life Sciences (Piscataway, NJ).

### Zinc-influx assay

We adapted a protocol from the one employed by Inoue and colleagues to monitor Zn^2+^-influx by TRPM7^22^. Briefly, TRPM7^−/−^ HEK293T cells were transiently transfected with pcDNA3.1D/V5-His-TOPO-XTRPM6, pcDNA3.1D/V5-His-TOPO-XTRPM7, pcDNA5/FRT/TO-mTRPM7, or pcDNA5/FRT/TO-hTRPM6 and allowed to express for 24–48 hours. The cells were labeled with the zinc indicator Fluo-Zin-3 following manufacturer’s orders (Thermo Fisher Scientific). The cells were then incubated with HBSS containing 30 μM ZnCl_2_ in the presence or absence of 250 μM 2-APB for 5 minutes, and then images were acquired on an inverted Olympus IX70 fluorescence microscope. The fluorescence intensity of the cells was analyzed using the Fiji software package with ROI analysis^32^.

### Statistical analysis

Statistical analysis used Student’s t-test. A p value of less than 0.05 was considered significant. Error bars indicate standard error.

### Data availability

No datasets were generated or analyzed during the current study.

## Electronic supplementary material

Supplementary Information
