# Supplementary material for: A Nonredundant Role for the TRPM6 Channel in Neural Tube Closure
Source: Sci Rep. 2017 Nov 15;7:15623. doi: 10.1038/s41598-017-15855-y (PMC5688082; doi:10.1038/s41598-017-15855-y)
Supplement: Supplementary file 1 — Supplementary Information [file 41598_2017_15855_MOESM1_ESM.pdf]

A Nonredundant Role for the TRPM6 Channel in Neural Tube Closure

Yuko Komiya[1,*],Zhiyong Bai[1], Na Cai[1], Liping Lou[1], Namariq Al-Saadi[1], Courtney Mezzacappa[2], Raymond Habas[2,*] & Loren W. Runnels[1,*].

[1]Rutgers-Robert Wood Johnson Medical School, Dept. of Pharmacology, Piscataway, 08854, U.S.A. [2]Temple University, Dept. of Biology, Philadelphia, 19122, U.S.A.
* runnellw@rwjms.rutgers.edu, habas@temple.edu, yukomiya1898@rs.tus.ac.jp

Supplementary Information

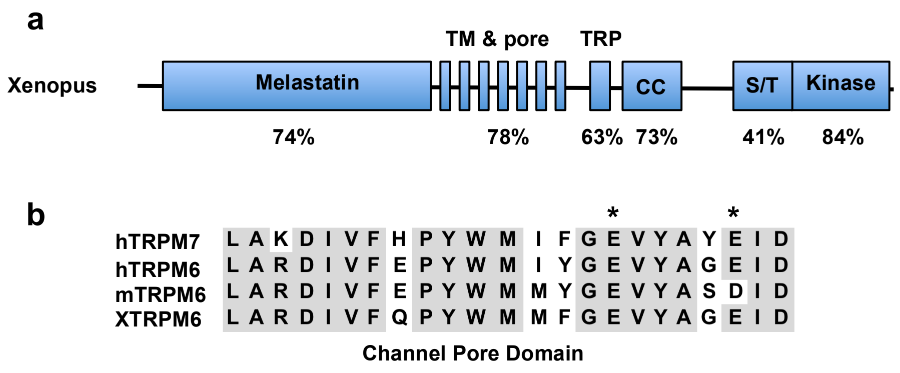

Supplementary Figure 1. Domain organization of XTRPM6 and alignment. (**a**) Schematic domain structure of XTRPM6. TM: transmembrane, CC: coiled coil, S/T serine-threonine rich region. The percentage sequence identity of each domain of XTRPM6 with human TRPM6 is shown. (**b**) Alignment of pore regions of TRPM6 orthologues with human TRPM7. The two glutamates that are determinants of hTRPM7's divalent selectivity are indicated with asterisks.

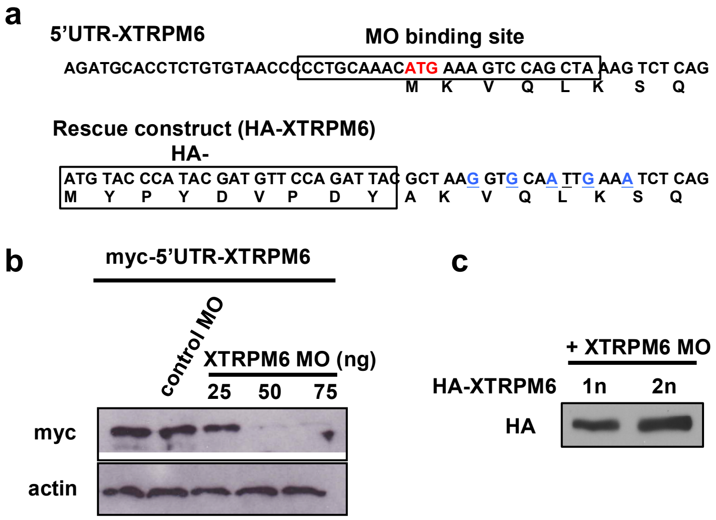

Supplementary Figure 2. TRPM6 MO efficiently blocks protein translation. (**a**) The sequence of Xenopus TRPM6 and HA-tagged XTRPM6 rescue constructs. XTRPM6 antisense MO sequence and HA-tagged sequence are indicated by boxes. Underlined and blue letters indicate mismatch bases between XTRPM6 and rescue construct. (**b**) XTRPM6 MO blocks translation of the Myc-tagged-5-'UTR-TRPM6 protein. Myc-tagged 5'UTR-XTRPM6 RNA was injected with control MO or XTRPM6 MO, and the amount of tagged protein was examined by Western blotting. (**c**) HA-tagged XTRPM6 (rescue construct) expression was not inhibited by the XTRPM6 MO. HA-tagged XTRPM6 RNA was injected with XTRPM6 MO and amount of tagged protein was examined by Western blotting.

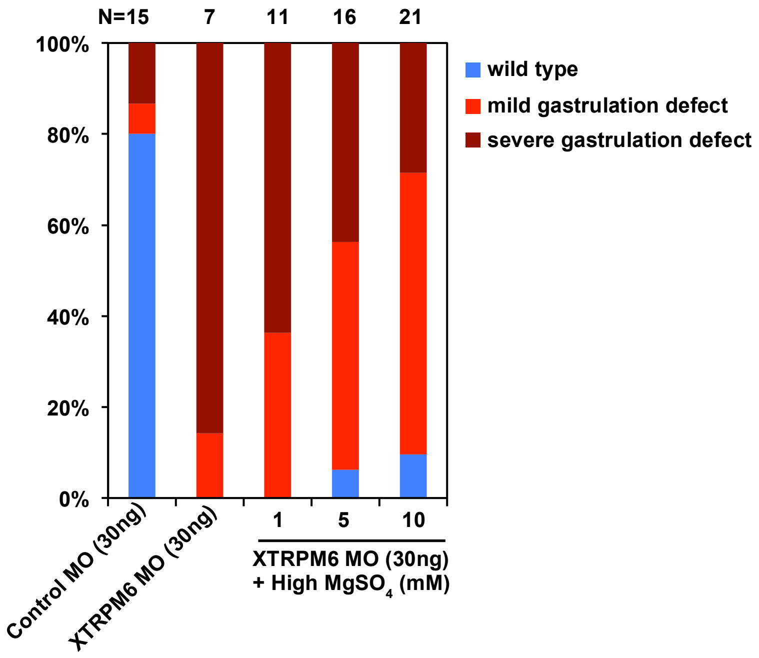

Supplementary Figure 3. Mg^{2+} supplementation suppressed TRPM6-MO-dependent gastrulation defects. Control MO or XTRPM6 MO was injected into the two dorsal blastomeres at the 4-cell stage and the phenotype was observed at tadpole stages. Addition of indicated concentration of $MgSO_4$ to 0.1 X Marc's Modified Ringer Buffer (10 mM NaCl, 0.2 mM KCl 0.1 mM $MgCl_2$, 0.2 mM $CaCl_2$, 0.5 mM HEPES, pH 7.5) used for culturing the embryos.

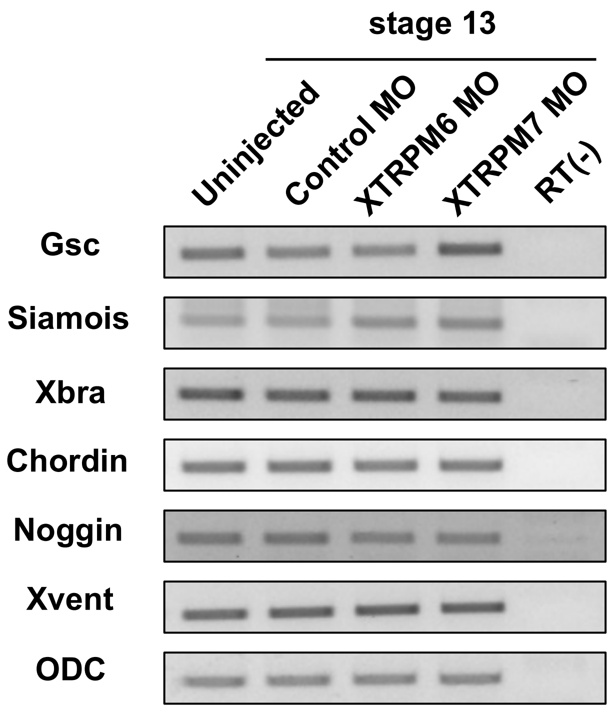

Supplementary Figure 4. XTRPM6 does not affect mesodermal specification. MOs were injected into two dorsal blastomeres at the 4-cell stage, and RNA was extracted at late gastrula stage (stage 13). RT-PCR was performed to monitor RNA levels of the indicated mesodermal genes.

ODC

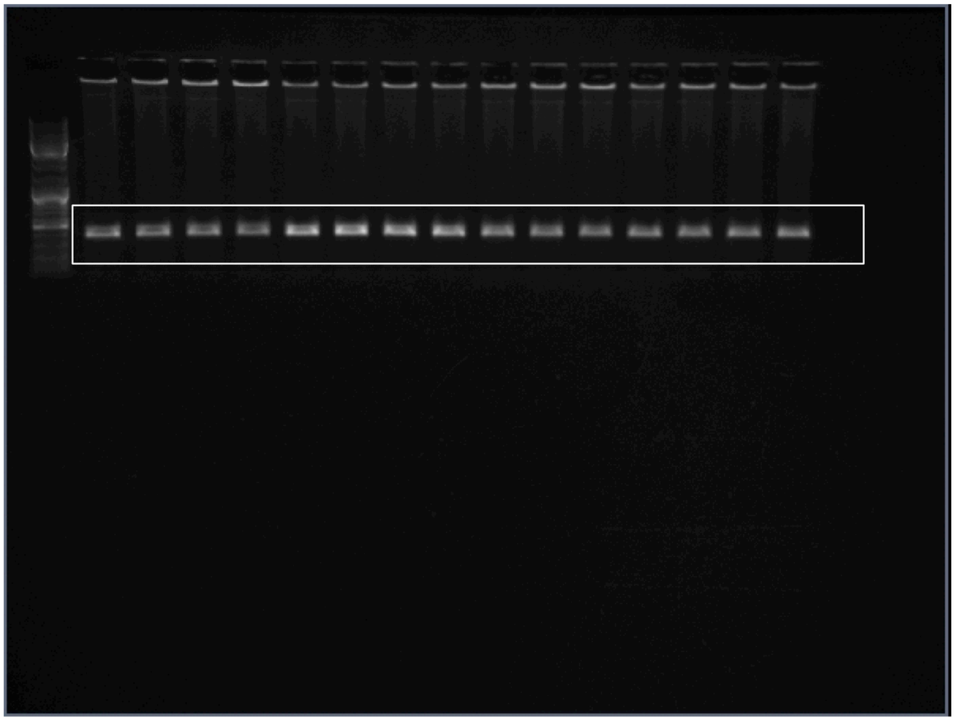

XTRPM6

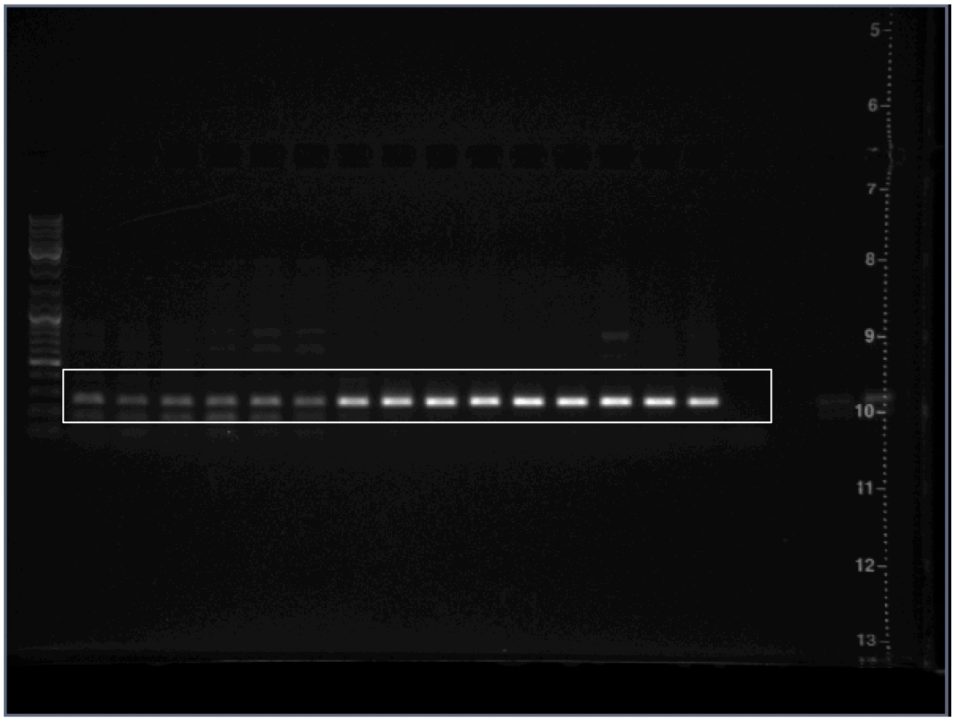

Supplementary Figure 5. Temporal expression pattern of XTRPM6. Full gel images of Figure 1a. White box indicates where images were cropped. The expression level of XTRPM6 RNA was assessed by RT-PCR analysis. ODC was used as an internal control. RT(-) is without reverse transcriptase as a negative control.
